# Multidrug resistant *Acinetobacter baumannii*: a descriptive study in a city hospital

**DOI:** 10.1186/1471-2334-10-196

**Published:** 2010-07-07

**Authors:** Lemuel L Dent, Dana R Marshall, Siddharth Pratap, Robert B Hulette

**Affiliations:** 1 Department of Surgery, Meharry Medical College, Nashville, USA; 2 Microarray and Bioinformatics Core, Meharry Medical College, Nashville, USA; 3 Department of Infection Control, Nashville General Hospital, Nashville, USA

## Abstract

**Background:**

Multidrug resistant *Acinetobacter baumannii*, (MRAB) is an important cause of hospital acquired infection. The purpose of this study is to determine the risk factors for MRAB in a city hospital patient population.

**Methods:**

This study is a retrospective review of a city hospital epidemiology data base and includes 247 isolates of Acinetobacter baumannii (AB) from 164 patients. Multidrug resistant *Acinetobacter baumannii *was defined as resistance to more than three classes of antibiotics. Using the non-MRAB isolates as the control group, the risk factors for the acquisition of MRAB were determined.

**Results:**

Of the 247 AB isolates 72% (177) were multidrug resistant. Fifty-eight percent (143/247) of isolates were highly resistant (resistant to imipenem, amikacin, and ampicillin-sulbactam). Of the 37 patients who died with Acinetobacter colonization/infection, 32 (86%) patients had the organism recovered from the respiratory tract. The factors which were found to be significantly associated (p ≤ 0.05) with multidrug resistance include the recovery of AB from multiple sites, mechanical ventilation, previous antibiotic exposure, and the presence of neurologic impairment. Multidrug resistant Acinetobacter was associated with significant mortality when compared with sensitive strains (p ≤ 0.01). When surgical patients (N = 75) were considered separately, mechanical ventilation and multiple isolates remained the factors significantly associated with the development of multidrug resistant Acinetobacter. Among surgical patients 46/75 (61%) grew a multidrug resistant strain of AB and 37/75 (40%) were resistant to all commonly used antibiotics including aminoglycosides, cephalosporins, carbepenems, extended spectrum penicillins, and quinolones. Thirty-five percent of the surgical patients had AB cultured from multiple sites and 57% of the Acinetobacter isolates were associated with a co-infecting organism, usually a Staphylococcus or Pseudomonas. As in medical patients, the isolation of Acinetobacter from multiple sites and the need for mechanical ventilation were significantly associated with the development of MRAB.

**Conclusions:**

The factors significantly associated with MRAB in both the general patient population and surgical patients were mechanical ventilation and the recovery of Acinetobacter from multiple anatomic sites. Previous antibiotic use and neurologic impairment were significant factors in medical patients. Colonization or infection with MRAB is associated with increased mortality.

## Background

*Acinetobacter baumannii*, found ubiquitously in the environment, is an aerobic gram negative rod which is a non-fermenter of glucose. When stationary, AB appears as a coccobacillus, however during growth it takes on a rod form. Multidrug resistant *Acinetobacter baumannii *is an important cause of hospital acquired infection and has been shown in some studies to increase mortality and length of stay [1].

Multidrug Resistant *Acinetobacter baumannii *is often associated with co-infection by other virulent pathogens. Thus it is difficult to determine its attributable mortality. Though MRAB is considered to be a hospital acquired infection, patients occasionally present with community acquired colonization of chronic wounds. In order to provide timely and proper antibiotic therapy it is important to know the characteristics of those patients with colonization and invasive infection with MRAB. The purpose of this study is to determine the resistance patterns of AB in a city hospital and to examine the risk factors for colonization/infection in surgical patients. The source of infection and the prevalence of co-infecting pathogens will also be investigated.

## Methods

### Setting

This research was approved by Institutional Review Board of Meharry Medical College. The Nashville General Hospital (NGH) is a 200 bed teaching tertiary safety net hospital which serves a population of 1.5 million people. NGH also serves as a major provider of inpatient care for the Tennessee Department of Corrections. The medical/surgical intensive care unit has 18 beds and is a combined general medical and surgical semi-closed unit.

### Study design

This study is a retrospective review of a city hospital epidemiology data base and includes 247 isolates of AB from 164 patients. For the period 2006 through 2008 a retrospective chart review was performed on all patients with AB isolates. The patients were identified through the hospital infection control data base. Documented patient demographics and potential risk factors included diagnosis, length of stay, patient location, age, sex, race, previous institutionalization, previous antibiotic use, mechanical ventilation, tracheostomy, and underlying co-morbidity. The source of the AB isolates was also noted as well as the patient outcome.

Previous institutionalization included prior hospitalization, nursing home residency, or incarceration within 90 days of a positive AB culture date. Prior antibiotic use was noted for those patients who received an antibiotic within one month of the AB isolation. The resistance patterns of all isolates were included in the analysis; however patients with more than one isolate were counted only once. The source of the AB isolate is the anatomic site where the culture was obtained.

### Identification and characterization of MRAB

The identification of the isolates as Acinetobacter and susceptibility testing of those isolates was performed using a Siemens (formerly Dade Behering) microscan system, which is FDA approved for this use in clinical laboratories. Testing was performed according to manufacturer specifications for this instrument and was done in accordance with NCCLS recommended practices. This instrument makes use of broth microdilution methodology to determine resistance. An isolate was deemed pan-sensitive if it was sensitive to all commonly tested antibiotics except colistin and highly sensitive if it was sensitive to imipenem, amikacin, and ampicillin-sulbactam. The isolate was considered highly resistant if it was resistant to imipenem, amikacin, and ampicillin-sulbactam. An isolate was classified as pan-resistant if it was resistant to all commonly used antibiotics. Multidrug resistant *Acinetobacter baumannii *isolates are defined as those resistant to more than three classes of antibiotics. A distinction was made between carbepenems and the non-carbepenem β-lactam antibiotics because carbapenem resistance is a sentinel event for emerging antimicrobial resistance and in itself confers high resistance. This study is designed to document the risk factors for the isolation of MRAB in our surgical patient population, thus no attempt was made to distinguish between colonization and invasive infection. The differences between groups were determined by Student's T-test for continuous data or the Fisher's exact test for categorical data (2-tailed). The significance level threshold was a p-value of ≤ 0.05.

## Results

Multidrug resistant *Acinetobacter baumannii *is defined as resistance to more than three classes of antibiotics. Of the 247 AB isolates, 177 (72%) were multidrug resistant. More than half of the isolates, 143/247 (58%) were resistant to imipenem, amikacin, and ampicillin-sulbactam; thus classifying these isolates as highly resistant. This is intriguing as these antibiotics were formerly very effective against AB. Only 42 (17%) of the isolates were sensitive to all three of the above antimicrobial agents. Forty-six percent (113/247) of the isolates were resistant to all commonly used antibiotics including aminoglycosides, cephalosporins, carbepenems, extended spectrum penicillins, and quinolones and therefore were classified as pan-resistant.

The sources of AB isolates in all patients are shown in Table 1. The major site of AB isolation in this study was the respiratory tract. Due to the inconsistency of obtaining quantitative cultures it was not possible to determine infection vs. colonization, however 32/37 (86%) of all patients who died had a positive isolate recovered from the respiratory tract. Among the positive wound isolates the majority were from chronic diabetic wounds, amputation sites, and decubitus ulcers. Using the non-MRAB isolates as the control group, the risk factors for the acquisition of MRAB were determined (Table 2). The factors which were significantly associated with multidrug resistance include the recovery of Acinetobacter from multiple sites, mechanical ventilation, previous antibiotic use, and the presence of co-morbidity (especially neurologic impairment). Diabetes mellitus and the previous use of quinolones trended toward significance.

**Table 1:** Sources of 247 multi-resistant *Acinetobacter baumannii *isolates

	N	%
Sputum	77	31

Urine	40	16

Extremity Wounds	32	13

Other	30	12

Blood	26	10

CVP Catheter	23	9

Decubitus Ulcer	19	8

**Table 2:** Risk factors for 247 isolates of multi-resistant *Acinetobacter baumannii *(all patients, N = 164)

Risk factor	MDRAB	Control	**p-value**^**1**^	Odds	**95% C.I**^**2**^
	(N = 122)	(N = 42)		Ratio	
Surgical admission	53	22	0.32	0.70	0.35-1.141

Age > 55	49	12	0.18	1.68	0.78 - 3.59

Male Gender	90	27	0.24	1.56	0.74 - 3.30

Institutionalized < 90 days prior to admission	91	32	0.84	0.92	0.41 - 2.08

Multiple Isolates	53	3	< 0.01	9.98	2.93-34.08

Mechanical Ventilation	65	3	< 0.01	14.82	4.35 - 50.57

Previous antibiotics	111	33	0.03	2.75	1.05 - 7.21

Quinolone	50	11	0.09	1.96	0.90 - 4.26

Co-morbidity^3^	111	33	0.03	2.75	1.05-7.21

COPD^4^	19	2	0.07	3.69	0.82 - 16.57

Neurologic impairment^5^	27	3	0.03	3.69	1.06 - 12.89

Diabetes mellitus	35	19	0.05	0.49	0.24 - 1.00

Mortality	36	1	< 0.01	17.24	1.70 - 83.33

^1 ^p-values derived from Fisher's exact test

^2 ^C.I. - 95% Confidence Interval

^3 ^Congestive heart failure, chronic pancreatitis, peripheral vascular disease, coronary artery disease, chronic obstructive pulmonary disease, diabetes mellitus, end stage renal disease, asthma, cancer, obesity, hepatitis, human immunodeficiency virus

^4^COPD - Chronic Obstructive Pulmonary Disease

^5^Cerebrovascular accident, Traumatic brain injury, Alzheimer's disease, Spinal cord injury

Forty six percent of the total patients (75/164) were admitted to the surgical service and the analysis appears in Table 3. The sites of AB isolation in surgical patients were wounds 36 (47.4%), respiratory tract 17 (23%), urinary tract 14 (18%), blood 5 (7%) and vascular catheters 3 (4%). Thirty-five percent of the surgical patients had AB cultured from multiple sites and 57% percent of the AB isolates in surgical patients were associated with a co-infecting organism, usually a Staphylococcus or Pseudomonas (Table 4). While the finding of Acinetobacter in multiple sites was associated with increased likelihood of multidrug resistance (p ≤ 0.01), no significant association was found for co-infecting organisms.

**Table 3:** Risk Factors for multi-resistant *Acinetobacter baumannii *in surgical patients (N = 75)

Risk factor	**MDRAB**^**1**^	Control	**p-value**^**2**^	Odds	**95% C.I.**^**3**^
	(N = 51)	(N = 24)		Ratio	
Age > 55	19	9	0.37	1.56	0.59-4.17

Male Gender	33	19	0.57	1.34	0.49 - 3.63

Institutionalized < 90 days prior to admission	32	20	0.96	1.03	0.38 - 2.82

Multiple Isolates	22	5	< 0.01	4.40	1.43 - 13.54

Mechanical Ventilation	22	4	< 0.01	5.73	1.72 - 19.09

Previous Antibiotics	41	25	0.73	1.31	0.32 - 5.35

Co-infection	26	8	0.64	1.09	0.76 - 1.56

Co-morbidity	41	23	0.32	2.14	0.59 - 7.79

Mortality	12	0	0.01	1.85	1.48 - 2.33

^1^Multi-resistant *Acinetobacter baumannii*

^2 ^p-values derived from Fisher's exact test

^3^C.I. - 95% Confidence interval

**Table 4:** Co-infecting organisms in 75 surgical patients

Organism	N	%
MRSA^1^	18	24

MSSA^2^	25	33

Pseudomonas aeruginosa	11	14

Proteus mirabilis	6	8

Escherichia coli	5	7

Enterococcus faecalis	5	7

Enterobacter cloacae	5	7

Staphylococcus epidermidis	4	5

Klebsiella pneumoniae	4	5

^1^Methicillin resistant *Staphylococcus aureus*

^2^Methicillin sensitive *Staphylococcus aureus*

Among surgical patients 46/75 (61%) of isolates were MRAB and 37/75 (40%) were resistant to all commonly used antibiotics. The majority of surgical patients with positive AB isolates (69%) had been institutionalized as inpatients, nursing home residents, or inmates within 90 days prior to the positive AB culture. Peculiar to this hospital population, 18 (24%) of surgical patients had been incarcerated. Three patients (4%) had Acinetobacter recovered from chronic wounds without a history of prior institutionalization. Twenty six surgical patients (35%) required mechanical ventilation or tracheostomy. Mechanical ventilation (p < 0.01) was significantly associated with the development of MRAB.

The majority (92%) of surgical patients with AB colonization received antibiotics within 30 days of the positive culture. The most frequent antibiotics prescribed were non-carbepenem β-Lactams (39%), and flouroquinolones (27%), aminoglycosides (15%), and carbepenems (13%). Overall prior exposure to antibiotics was not a significant risk factor in the development of MRAB in this group of surgical patients, however the previous use of non-carbepenem β-lactams trended toward significance (p = 0.07).

A co-morbidity was present in 63(84%) of surgical patients but overall was not a risk factor for MRAB in this study (p = 0.32). The most frequent co-morbidity associated with MRAB in this study is diabetes mellitus 27/75 (36%), followed by chronic obstructive pulmonary disease (12%), neurological impairment (9%), and cardiovascular disease (8%).

With regard to outcome, 41% of patients were discharged to home, 32% were discharged to nursing homes or transferred to other inpatient facilities, and 16% died. The mean length of stay for sensitive and multidrug resistant Acinetobacter (32 vs. 29 days) was not significantly different (p>0.05). Of the surgical deaths, all patients had MRAB and 9/12(75%) were colonized or infected with pan-resistant AB. Overall, the presence of co-infection with other organisms did not contribute significantly to mortality (p = 0.75). The only organism that approached significance with respect to affecting survival was *Pseudomonas aeruginosa *(p = 0.07).

## Discussion

*Acinetobacter baumannii *has become an important pathogen in recent years and has been shown to increase morbidity and mortality [1-3]. The definition of MRAB varies in the literature, but several authorities consider an isolate to be multidrug resistant if it is resistant to three or more classes of antibiotics [4]. Resistant AB is a significant problem as seen in this study where 72% of isolates were considered multidrug resistant and 58% were resistant to imipenem, amikacin, and ampicillin-sulbactam, formerly very effective antibiotics. Nearly half (46%) of all isolates were resistant to all commonly used antibiotics including aminoglycosides, cephalosporins, carbepenems, extended spectrum penicillins, and quinolones. A distinction was made between carbepenems and the non-carbepenem β-lactam antibiotics because carbapenem resistance is a sentinel event for emerging antimicrobial resistance and in itself confers high resistance and therapeutic challenges [5].

The factors associated with the isolation of AB in our combined medical and surgical patient groups include mechanical ventilation, previous antibiotic therapy, co-morbidity, especially neurologic impairment, and multiple Acinetobacter isolates. The significant factors for MRAB in the surgical group were mechanical ventilation, multiple isolates, and neurologic impairment. These findings are consistent with other reports [6-9]. The most frequent antibiotics prescribed in this study population were β-lactams, followed by flouroquinolones, aminoglycosides, and carbepenems. The prior exposure to quinolones trended toward significance which is documented in previous reports [10].

Several investigators have found an association of MRAB with co-morbidities [7]. Our patients had significant rates of diabetes mellitus, cardiovascular disease, chronic obstructive lung disease and neurological impairment. A co-morbid condition was found in 84% of our surgical cohort, however only neurologic impairment achieved significance. Neurologic injury, in particular paraplegia has been shown to be associated with the development of resistant Acinetobacter [11]. Our neurologically impaired population had a high prevalence of chronic wounds such as decubitus ulcers and most were nursing home residents. This most likely explains the significant increased risk of resistant Acinetobacter in this group.

Chronic obstructive pulmonary disease and diabetes showed a trend toward significant associations with the development of multidrug resistant Acinetobacter. It is well accepted that patients with chronic lung disease are at increased risk of airway colonization and pneumonia, especially when they require intubation [12]. Additionally, intubated patients with chronic pulmonary disease are often treated with prophylactic antibiotics which increase the risk of resistance. The association with diabetes is most likely related to the high prevalence of chronic diabetic lower extremity wounds in this patient population.

As in several other studies, this descriptive study attests to the ability of AB to colonize/infect soft tissue and bone [13,14]. Nearly half (47%) of our surgical patients had resistant AB in chronic diabetic wounds, amputation sites, or decubitus ulcers. Several our patients had multiple positive cultures of their wounds over a period of several months. These patients are chronic carriers of resistant AB who may develop life threatening invasive infection or serve as portals of entry of resistant AB into the hospital. Whether routine surveillance and isolation of these patients may help to prevent outbreaks is unclear [11].

Previous institutionalization (hospital, prison, or nursing home) did not reach the level of significance (p = 0.07) as a factor in the development of MRAB in this study. The reason for this finding is unclear since several studies have documented higher rates of MRAB in patients with a history of prior institutionalization [8]. Our patient population is unusual due to the large number (24%) of incarcerated individuals. Three patients (4%) had multidrug resistant Acinetobacter despite having no history of being institutionalized. The existence of community acquired MRAB strains would present significant challenges to infection control.

As seen in Tables 1 and 2, MRAB was associated with a significant (p < 0.01) increase in mortality. An increase in mortality for ventilator associated Acinetobacter pneumonia has been noted in several studies [2,15,16], however other investigators have not confirmed this finding [17,18]. In the surgical group, a significant proportion (35%) had positive Acinetobacter cultures from multiple sites. Multiple isolates were associated with a significant increase in mortality. The finding of resistant AB isolates in more than one anatomic site most likely signifies an invasive infection rather than colonization. The majority of surgical patients (57%) had an organism other than AB identified on culture, usually a Staphylococcus or Pseudomonas. Co-infection with non-Acinetobacter organisms did not lead to a significant increase in mortality. Pseudomonas was the only organism that approached significance with respect to mortality (p = 0.07)

There was no significant difference in the length of stay between patients with sensitive isolates vs. multidrug resistant Acinetobacter. This finding was somewhat surprising given that some studies have shown an increase length of stay for resistant strains. The length of stay in patients with resistant organisms may be confounded by their increased mortality. The mortality rate in surgical patients was 16% and all had resistant Acinetobacter. Most of the surgical patients who died (75%) grew pan-resistant strains which were resistant to all commonly used antibiotics. Underlying co-morbidities and co-infecting organisms did not significantly impact mortality in this study, whereas the presence of a multidrug resistant Acinetobacter was associated with increased mortality (OR 1.6, 95% C.I 1.33-1.96). This suggests that colonization/infection with MRAB is a marker for severe illness or that the organism itself is responsible for poor outcome.

This study is limited because it does not identify patients with true infections versus those that are colonized. The goal was to determine the impact of MRAB in our hospital. The mortality rate in this study of 16% is consistent with a similar study reported by Mahgoub [9]. Of note is that the rate of MRAB in our hospital was 122/164 (75%) which is higher than reported elsewhere [19]. The cause of the high rate of multi-resistance is not known and our study design does not enable us to answer this question. We are not certain what role our infection control procedures had on the high resistance rate. Standard infection control practices were followed (hand hygiene, personal protective equipment, environmental control, isolation, etc). Most of the resistant cases (75%) occurred in the ICU, however antibiotic susceptibilities and the anatomical sources varied greatly. We speculate that since different strains were involved, it is unlikely that a breakdown in infection control was the major cause of the high rate of resistance. Initially, the multi-resistant isolates were confirmed by an independent laboratory. Thus, we are confident concerning our determination of the antibiotic susceptibilities. We are planning a future study to determine the DNA fingerprinting of several of the more common resistant phenotypes in our hospital.

## Conclusions

The factors associated with multidrug resistant isolates in this study were mechanical ventilation, previous antibiotic use, neurologic impairment, and the recovery of Acinetobacter from more than one anatomic site. This study also suggests that infection or colonization with MRAB is associated with increased mortality. The increasing presence of MRAB in wounds is particularly problematic in the surgical patient. Chronic wounds such as amputation stumps, decubitus ulcers, and diabetic wounds are portals of entry of Acinetobacter into the surgical intensive care unit. A small number of patients present with community acquired multidrug resistant strains, thus routine surveillance may be useful to guide hospital infection control measures. Vigilance is needed by the surgical team to prevent outbreaks of this opportunistic and deadly pathogen.

## Competing interests

The authors declare that they have no competing interests.

## Authors' contributions

LD designed the study, collected and analyzed the data, and drafted the manuscript. DM assisted with study design, collection of data, revised and approved the manuscript. SP provided statistical support, assisted with study design, revised and approved manuscript. BH provided culture data, data interpretation, reviewed and approved the manuscript.

## Pre-publication history

The pre-publication history for this paper can be accessed here:

http://www.biomedcentral.com/1471-2334/10/196/prepub
